# *Pseudomonas fluorescens* increases mycorrhization and modulates expression of antifungal defense response genes in roots of aspen seedlings

**DOI:** 10.1186/s12870-018-1610-0

**Published:** 2019-01-03

**Authors:** Shalaka Shinde, Sarah Zerbs, Frank R. Collart, Jonathan R. Cumming, Philippe Noirot, Peter E. Larsen

**Affiliations:** 1 Argonne National Laboratory, Biosciences Division, 9700 S. Cass Ave., Argonne, IL 60439 USA; 2 Department of Biology, West Virginia University, 53 Campus Dr, Morgantown, WV 26506 USA; 3 Department of Bioengineering, University of Illinois at Chicago, 851 South Morgan St., Chicago, IL 60612 USA; 4 Present address: Oil-Dri Innovation Center, 777 Forest Edge Rd., Vernon Hills, IL 60061 USA

**Keywords:** Ectomycorrhiza, *Laccaria bicolor*, Mycorrhiza helper bacteria, *Populus tremuloides*, Receptors, Transcriptomics

## Abstract

**Background:**

Plants, fungi, and bacteria form complex, mutually-beneficial communities within the soil environment. In return for photosynthetically derived sugars in the form of exudates from plant roots, the microbial symbionts in these rhizosphere communities provide their host plants access to otherwise inaccessible nutrients in soils and help defend the plant against biotic and abiotic stresses. One role that bacteria may play in these communities is that of Mycorrhizal Helper Bacteria (MHB). MHB are bacteria that facilitate the interactions between plant roots and symbiotic mycorrhizal fungi and, while the effects of MHB on the formation of plant-fungal symbiosis and on plant health have been well documented, the specific molecular mechanisms by which MHB drive gene regulation in plant roots leading to these benefits remain largely uncharacterized.

**Results:**

Here, we investigate the effects of the bacterium *Pseudomonas fluorescens* SBW25 (SBW25) on aspen root transcriptome using a tripartite laboratory community comprised of *Populus tremuloides* (aspen) seedlings and the ectomycorrhizal fungus *Laccaria bicolor* (*Laccaria*). We show that SBW25 has MHB activity and promotes mycorrhization of aspen roots by *Laccaria*. Using transcriptomic analysis of aspen roots under multiple community compositions, we identify clusters of co-regulated genes associated with mycorrhization, the presence of SBW25, and MHB-associated functions, and we generate a combinatorial logic network that links causal relationships in observed patterns of gene expression in aspen seedling roots in a single Boolean circuit diagram. The predicted regulatory circuit is used to infer regulatory mechanisms associated with MHB activity.

**Conclusions:**

In our laboratory conditions, SBW25 increases the ability of *Laccaria* to form ectomycorrhizal interactions with aspen seedling roots through the suppression of aspen root antifungal defense responses. Analysis of transcriptomic data identifies that potential molecular mechanisms in aspen roots that respond to MHB activity are proteins with homology to pollen recognition sensors. Pollen recognition sensors integrate multiple environmental signals to down-regulate pollenization-associated gene clusters, making proteins with homology to this system an excellent fit for a predicted mechanism that integrates information from the rhizosphere to down-regulate antifungal defense response genes in the root. These results provide a deeper understanding of aspen gene regulation in response to MHB and suggest additional, hypothesis-driven biological experiments to validate putative molecular mechanisms of MHB activity in the aspen-*Laccaria* ectomycorrhizal symbiosis.

**Electronic supplementary material:**

The online version of this article (10.1186/s12870-018-1610-0) contains supplementary material, which is available to authorized users.

## Background

Terrestrial plants are rarely solitary organisms. Rather, plants form complex, mutually-beneficial communities with fungi and bacteria that live within the rhizosphere, which is the narrow band of soil directly infused by plant root exudates. Plants provide photosynthetically-fixed carbon, primarily in the form of exuded sugars and organic acids, to rhizosphere community members [1]. In return, the plant receives a wide variety of ecological services from its root-associated organisms, including access to sources of nutrients that would otherwise be unavailable to the plant and protection from biotic and abiotic stresses [2–5].These interactions occur through the exchange of small molecules, such as nutrients, signaling compounds, and small secreted proteins between the community partners [6–8].

One of the specific ecological services provided by bacteria in the rhizosphere is that of Mycorrhizal Helper Bacteria (MHB), a concept that was first introduced over 20 years ago by Garbaye [9]. MHB interact positively with plants and mycorrhizal fungi to enhance the functioning of the plant-fungal symbiosis [10, 11]. MHB activities have been observed across many bacterial groups, such as *Actinomycetes*, *Firmicutes*, and *Proteobacteria*, and with both ectomycorrhizal and arbuscular mycorrhizal symbioses [12]. Because of the diversity in both MHB species and possible fungal partners, including ectomycorrhizal fungi (e.g., [11]) and arbuscular mycorrhizal fungi (e.g., [13]), it is unlikely that any single biological mechanism can account for all MHB interactions. Rather, a number of general and non-exclusive mechanisms of MHB interactions have been proposed [10, 11, 14–16]. MHB can promote the germination of fungal spores in soil and promote mycelial growth, enhancing fungal presence in the rhizosphere [15]. MHB can condition the soil by removing toxins, antibiotics, or other compounds that inhibit fungal growth in the rhizosphere and plant-fungal interactions [15]. MHB can also secrete plant cell wall-digesting enzymes that make it easier to fungi to form symbiotic interactions [17]. More importantly, however, MHB facilitate plant-fungal interactions through the production of effectors and/or hormones that drive patterns of gene regulation in both plant roots and mycorrhizal fungi hyphae that enhance their subsequent mycorrhizal interactions [10, 18].

Extensive cross-talk between host roots, ECM fungi, and MHB bacterial symbionts is responsible for broad metabolic remodeling that leads to morphological and functional changes in the symbiotic root [14, 19, 20]. Underlying this communication are diffusible molecules and their sensors that translate these signals into regulatory outcomes. MHB may manipulate gene regulation in the fungus as a way to increase mycorrhization, for example through the production of stimulating flavonoids or hormones [19] to attract the mycorrhizal symbiont, alter of host innate immune responses, or through broad changes in fungal gene regulation shifting mycelium from free-living to pre-symbiotic states [2, 10].

While MHB is a well-known phenomenon [3] and the transcriptomic effects of bacteria on soil fungi has been investigated [10, 21], the transcriptomic regulatory interactions in plant roots that enable MHB activity are less well studied. Here, we focus on the MHB’s effect on aspen seedling root transcriptional profiles to gain insight into the possible adaptive responses of aspen roots to the presence of MHB. We utilize a tripartite laboratory system composed of *Populus tremuloides* (aspen) seedlings, the ectomycorrhizal fungus *Laccaria bicolor* (*Laccaria*), and the plant growth promoting (PGP) bacterium *Pseudomonas fluorescens* SBW25 (SBW25) for investigating the molecular mechanisms of MHB effects of aspen root gene regulation. The aspen-*Laccaria*-*Pseudomonas* system is an excellent laboratory model of rhizosphere community interaction, as *Laccaria* readily forms ectomycorrhizae with aspen roots in the laboratory. While, prior to this publication, there have been no direct reports of SBW25 specifically functioning as a MHB, the MHB activity of SBW25 is strongly anticipated. More than 80% of the *Pseudomonas* isolates collected from *Populus* rhizospheres had a positive effect on *Laccaria* growth [11]. SBW25 forms complex and dynamic biofilm structures on aspen roots [22]. Also, bacterial isolates from plant roots that have plant growth promoting (PGP) activities were also frequently found to stimulate mycelial growth and mycorrhizal formation [10, 11] and SBW25 has PGP effects for aspen seedlings, including under conditions of nitrogen and phosphorus nutrient limitation [23].

Through analysis of aspen seedling root transcriptome data collected from the tripartite laboratory communities of various compositions, we identify clusters of co-regulated genes that are associated with mycorrhization, the presence of SBW25, and MHB-related functions. Patterns of gene cluster expression across experimental conditions are assembled into predicted regulatory networks that highlight the importance of inhibition of antifungal response activities for MHB interactions and identify specific sensor proteins in aspen root that potentially enable MHB interactions.

## Results

### Phenotypic measurements of aspen in tripartite community

**Fig. 1:**
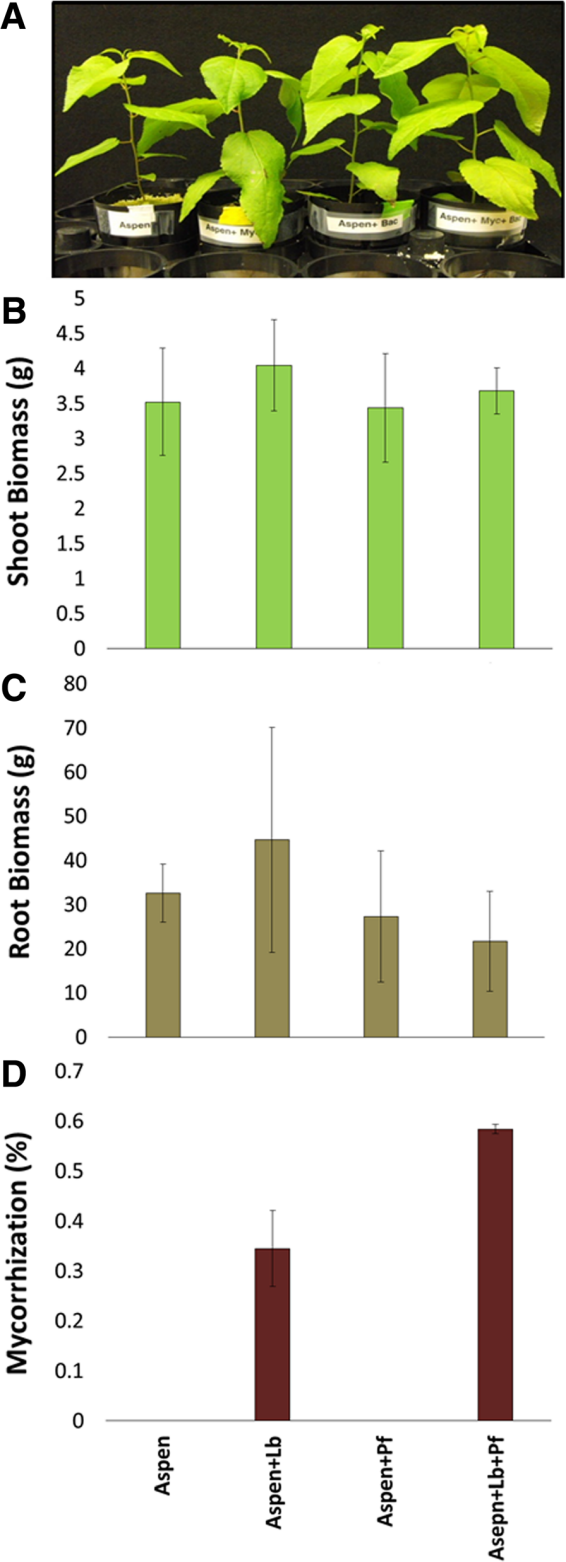
Tripartite community phenotypes. (**a**) Aspen seedlings were grown in sand pots in four experimental conditions: aspen seedlings alone (Aspen), aspen with the mycorrhizal fungi *Laccaria* (Aspen+Lb), aspen with the MHB *P. fluorescens* SBW25 (Aspen+Pf), and aspen with both *Laccaria* and SBW25 (Aspen+Lb + Pf). A typical example of aspen under each experimental condition after 63 days of growth is shown. (**b**) Shoot biomass, (**c**) Root biomass, and (**d**) Percent mycorrhization of aspen seedling roots were measured in tripartite community systems (‘Aspen’ = aspen, ‘Lb’ = *L. bicolor*, ‘Pf’ = *P. fluorescens*). ‘*’ indicates a statistically significant difference between Percent mycorrhization with *Laccaria* and with *Laccaria* + SBW25

Aspen seedlings were cultured in sand pots supplemented under four experimental conditions: aspen seedlings alone, aspen seedlings inoculated with *Laccaria*, aspen seedlings inoculated with SBW25, and aspen seedlings co-inoculated with both *Laccaria* and SBW25. For each condition, eight biological replicates were generated, of which five were used to measure seedling biomass and quantify root mycorrhization. The results of phenotypic analysis of tripartite mycorrhizal communities are summarized in Fig. 1. The presence of *Laccaria*, SBW25, or *Laccaria* plus SBW25 did not significantly affect either shoot or root biomass under our nutrient-replete laboratory experimental conditions. However, in the presence of SBW25, the percent proportion of aspen roots in mycorrhizal interaction by with *Laccaria* was significantly increased by 1.7-fold (two-tailed t-test *p*-value of 0.006).

### Distribution of aligned RNA-seq reads to aspen, *Laccaria*, and SBW25 gene sequences

Three aspen seedlings per condition were used as biological replicates to collect root transcriptome data (see Methods). The total number of sequence reads that aligned to the set of gene models for each community member is summarized in Additional file 1. Of the sequence reads that aligned to one of the tripartite community organisms, the great majority (> 99% on average) aligned to aspen gene sequences with the remaining reads aligning to either *Laccaria* or SBW25 gene sequences (an average of 1.4 and 0.005% of total aligned reads respectively). Because 50b-long RNA-seq data was used, finding some reads that align to ‘*Laccaria*’ or ‘SBW25’ gene sequences also aligning with aspen sequences data is unavoidable [24]. Thus, the total percent aligned sequenced reads attributed to community members may sum to more than 100% as some sequence reads will align to genes from more than one community member (Table S1).

To identify if *Laccaria* or SBW25 was present in the rhizosphere community from transcriptomic data, we determined whether a statistically significant enrichment for fungal or bacterial reads could be detected in transcriptomes where SBW25 or *Laccaria* are present *relative* to those experimental conditions where SBW25 or *Laccaria* are absent. When *Laccaria* was inoculated into the community, there is a 13.6-fold enrichment of *Laccaria*-aligned reads in the transcriptome (*p*-value 0.065). In samples for which SBW25 was inoculated into the community, there is a 2.6-fold enrichment for SBW25-aligned reads (p-value 0.078). When *Laccaria* and SBW25 were both present in the community, there is a significant 3.5-fold increase in bacterial reads in the tripartite community relative to the apsen-SBW25 culture condition (p-value 0.046), although the difference in *Laccaria* reads is not significantly different in this condition compared to the *Laccaria*-only community. These enrichments indicate that transcriptionally-active SBW25 and *Laccaria* cells were established and functional in the rhizosphere of our experimental system. Furthermore, SBW25 was present in either higher abundance or in more biologically active states (i.e. higher rates of transcription) when *Laccaria* was also present. A negative correlation (PCC = − 0.55) is observed between root biomass and percent RNAseq reads aligning to SBW25 genome suggesting that increased presence of SBW25 in the tripartite condition is not due to increased root biomass.

### Differential gene expression in the aspen root transcriptome

**Fig. 2:**
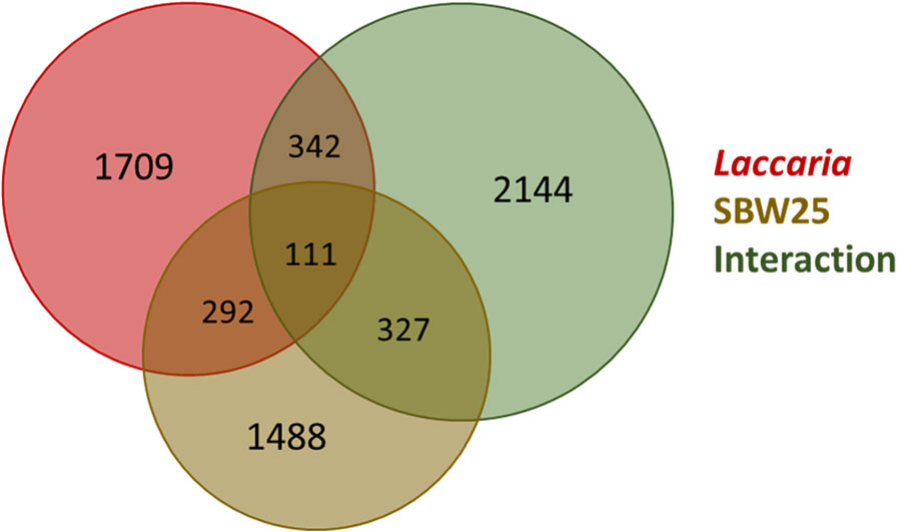
Venn diagram of differentially expressed genes by experimental factor. Differential expression in aspen root transcriptome was determined by 2-factor ANOVA. Factors in ANOVA were Presence/Absence of *Laccaria*, Presence/Absence of SBW25, and Interaction between *Laccaria* and SBW25

6413 aspen genes (15.5% of all aspen genes) were identified as significantly differentially expressed as a function of community composition by 2-factor ANOVA with FDR-corrected *p*-values less than 0.05 (Fig. 2). 2454 genes (7%) were differentially expressed in the presence of *Laccaria*, 2218 genes (7%) were differentially expressed in the presence of SBW25, and 2924 genes (9%) were differentially expressed due to the co-presence of *Laccaria* and SBW25. 403 genes (6.3% of all differentially expressed genes) were common between fungus and MHB conditions and 111 genes (1.7% of all differentially expressed genes) were common to *Laccaria*, SBW25, and co-presence conditions. All gene expression values, fold-change values, and condition identification can be found in Additional file 2**.**

### Co-regulated gene clusters

**Fig. 3:**
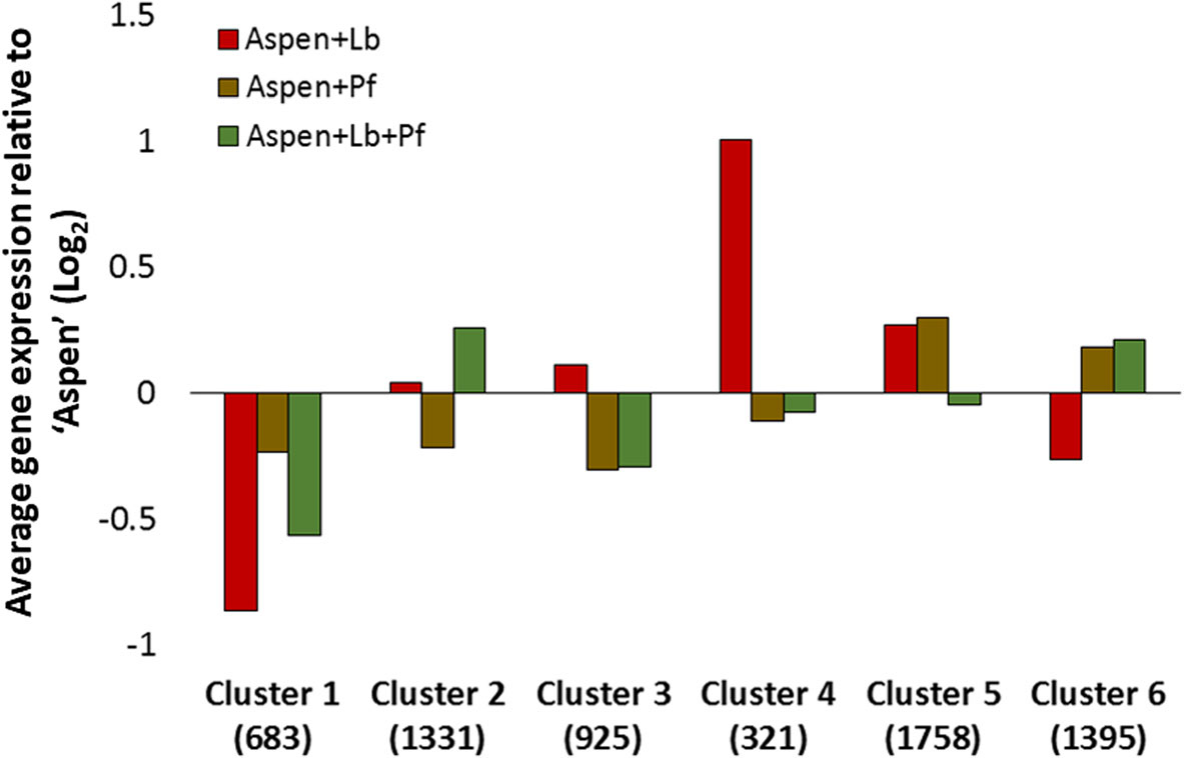
Average expression, relative to aspen-monoculture condition, for genes in co-regulated clusters. X-axis is labeled with co-regulated gene Cluster number. Number in parenthesis is the total number of genes in that cluster. Y-axis indicates the average log_2_ fold difference between all genes within a cluster, relative to the aspen-only condition. ‘Aspen’ = aspen, ‘Lb’ = *L. bicolor*, ‘Pf’ = *P. fluorescens*

**Fig. 4:**
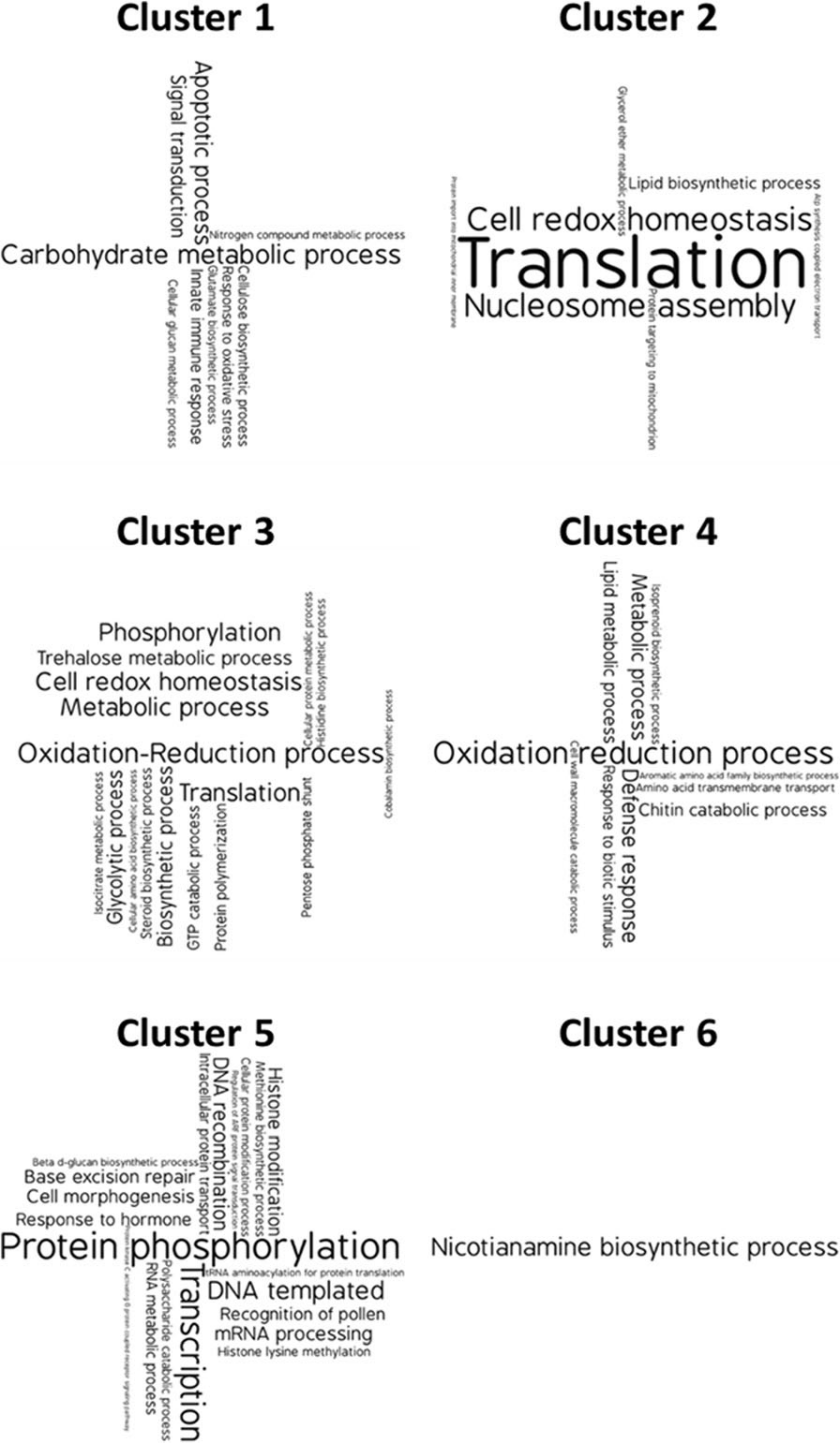
Enriched GO-BP annotations in clusters of co-expressed aspen root genes. Tag clouds of significantly enriched GO-BP annotation terms in each cluster of co-regulated genes are presented with the size of annotation tag proportionate to the number of genes with that annotation in the cluster. Figures generated using web-based WordArt tool (https://wordart.com/). Enriched annotations can be found in tabular format in Additional file 4

Significantly differentially-expressed aspen genes were grouped into 6 clusters of similar expression patterns using K-means clustering. The average expression of genes in each cluster relative to the average expression for aspen alone and the numbers of genes in each cluster is summarized in Fig. 3**.** The complete set of annotated genes, their cluster membership, and their expression levels can be found in Additional file 3. Gene Ontology Biological Function (GO-BF) annotations that were found to be statistically significantly enriched in each cluster, relative to the distribution of annotations in the aspen genome, are presented as a text clouds in Fig. 4 and the enriched GO-BF annotations can be found in tabular format in Additional file 4.

Genes in Cluster 1 are down-regulated in all co-culture conditions relative to aspen roots alone, but most dramatically down-regulated when aspen seedlings are co-cultured only with *Laccaria* (Fig. 3). Significantly enriched annotations in Cluster 1 are linked to the seedling’s innate immune detection and response: signal transduction, stress response, apoptotic process, and innate immune system as well as genes associated with biosynthesis of plant cell walls (glycan metabolism, carbohydrate metabolism, and cellulose biosynthesis) (Fig. 4).

Co-regulated genes in Cluster 2 are down-regulated when only SBW25 is present, slightly upregulated when only *Laccaria* is present, and more strongly up-regulated when SBW25 and *Laccaria* are both present (Fig. 3). This expression pattern strongly correlates with percent mycorrhization (PCC 0.99), and is significantly higher than the percent mycorrhizal correlation with any other gene cluster (average correlation is − 0.25, standard deviation is 0.45, significance *p*-value of 0.002 calculated using the Normal Distribution). Enriched annotations in this cluster are for regulation and global transcriptome reprograming activities (nucleosome assembly and a large set of translation-associated genes) (Fig. 4).

Co-regulated gene expression Clusters 3 and 6 are regulated only by the presence of SBW25 and are independent of the presence or absence of *Laccaria* (Fig. 3). Genes in Cluster 3 are down-regulated whenever SBW25 is present. Enriched annotations in this bacteria-specific gene cluster are associated with amino acid metabolism (histidine and amino acid biosynthesis), protein production and modification functions (translation, protein polymerization, and protein metabolic process), and biosynthesis of signaling molecules (steroid biosynthesis, isocitrate metabolism, and cobalamin biosynthesis) (Fig. 4). Regulated in the opposite direction as Cluster 3, genes in Cluster 6 are upregulated when SBW25 is present, but down-regulated when only *Laccaria* is present. Although Cluster 6 contains a large number of genes, the only significantly enriched annotations are associated with nicotiamine synthesis (Fig. 4), suggesting that this cluster may capture gene functions that are not specific for mycorrhizal or MHB interactions.

Co-regulated genes in Cluster 4 are associated with innate immune response and antifungal defense and are most strongly regulated when *Laccaria* alone is present in the mycorrhizal community (Fig. 3). Genes in Cluster 4 indicate that defense against pathogenic fungi (defense response, response to biotic stimulus, amino acid transport, and chitin catabolism) is highly up-regulated when only *Laccaria* is present. This pattern strongly anticorrelates with the expression pattern of innate immune and cell wall remodeling response gene Cluster 1 (PCC -0.76), which represents the most significant correlation among gene clusters (average correlation is − 0.13, standard deviation is 0.43).

Cluster 5 is the largest of the identified co-regulated gene clusters and significantly enriched gene annotations suggest the activation of a variety of signaling processes (signaling pathways, response to hormone, signal recognition, and a large number of protein phosphorylation genes) leading to gene regulation (histone modifications, tRNA aminoacylation) and transcription (transcription and mRNA processing) (Fig. 4).

### Patterns of regulation for gene clusters described as Boolean logic circuit

**Fig. 5:**
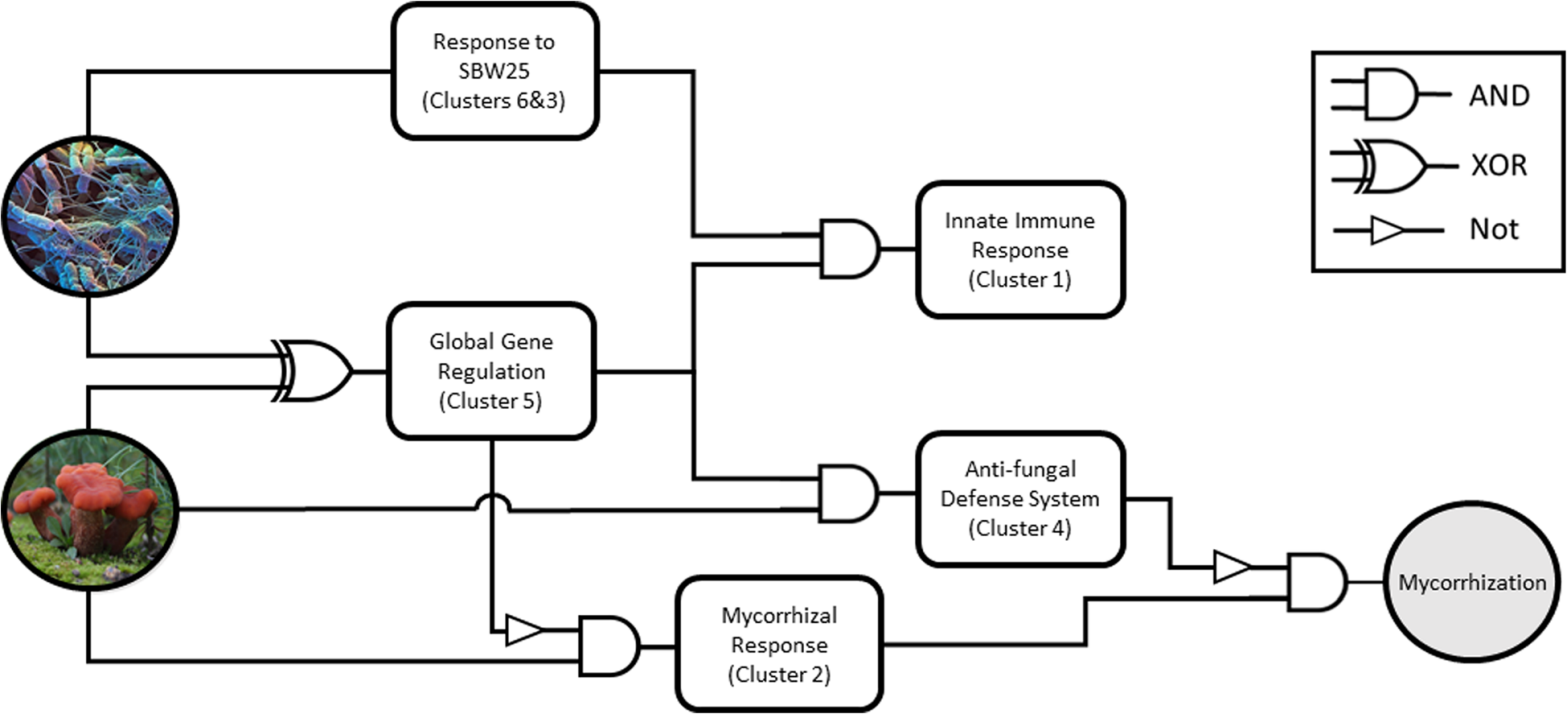
Predicted aspen root gene network for MHB activity. The proposed regulatory interactions between co-expressed gene clusters is represented as a logic circuit diagram. In the network, circles to left indicate presence or absence of *Laccaria* (red mushrooms) or SBW25 (blue microscopy image). Rectangles are co-regulated gene clusters. Cluster numbers reference cluster IDs as presented in Fig. 3 and cluster functions are draws from enriched gene annotations as presented in Fig. 4. Edges indicate predicted causal relationships between gene clusters inferred from observed patterns of gene regulation by Bayesian Inference analysis

By combining the patterns of co-regulated gene clusters and enriched annotations, above, with a Bayesian belief network of gene clusters and presence/absence of rhizosphere community members (see Methods), a Boolean logic circuit diagram that reflects causal links between the gene clusters and MHB activity was generated (Fig. 5).

In the Boolean circuit, the presence of either SBW25 or *Laccaria* alone activates the ‘Global Gene Regulation’ cluster (Cluster 5). The presence of SBW25 alone then also regulates the ‘Response to SBW25’ clusters (Clusters 3 and 6) and the ‘Innate Immune Response’ cluster (Cluster 1). The presence of *Laccaria* alone leads to inhibition of the ‘Mycorrhizal Response’ cluster (Cluster 2) and activation of the ‘Antifungal Defense System’ cluster (Cluster 4), which inhibits the ‘Mycorrhization’ cluster. The combined presence of both SBW25 and *Laccaria*, however, does not activate the ‘Global Gene Regulation’ cluster, which then allows the activation of ‘Mycorrhizal Response’ cluster, inhibits the ‘Antifungal Defense System’ cluster, and leads to MHB-enhanced mycorrhization of aspen roots (Fig. 5). The position of Cluster 5 in the circuit and the preponderance of regulatory-related function annotations noted above (Fig. 4) makes this cluster a likely target for aspen root regulatory elements most closely associated with MHB detection and subsequent gene regulation. The Bayesian belief network can be found in Additional file 5.

## Discussion

Forest trees rely extensively on mycorrhizal symbionts to access limiting soil nutrients in the environment. ECM fungi increase root surface area and contribute new metabolic capabilities to the host tree, all of which alter root-soil interactions. The formation of the ectomycorrhizal symbiosis requires remodeling of the host root immune system, allowing subsequent changes of root structure and function characteristic of fungal-host specific mycorrhizas. This interaction is the result of effectors produced by fungi, including the hormones auxin and ethylene and small secreted effector proteins, that alter host transcription and metabolism to facilitate the colonization of short roots by the ECM fungus [20]. Changes induced by fungal signals include stimulation of lateral root production as well as changes in cell wall metabolism that foster sites for mycorrhiza formation.

In addition to symbiotic mycorrhizal fungi, roots and mycorrhizas are colonized by a diverse bacterial community, many of which similarly alter host physiology and contribute novel metabolic pathways that foster resource acquisition and host environmental stress resistance [3, 25]. Further, interactions between microbial symbionts indicate that bacterial-fungal communication influences the ecological structure and function of the rhizosphere community and its interaction with plant roots, with MHB significantly increasing mycorrhization of tree roots [11, 18, 21]. While the molecular signals of some of these interactions have begun to be explored [10, 21, 26], significantly less attention has focused on host molecular response underpinning the tripartite interaction.

### *Pseudomonas fluorescens* SBW25 is a mycorrhizal helper bacterium

While the presence of SBW25 had a significant, positive effect on short root development and mycorrhization of aspen roots by *Laccaria*, neither *Laccaria* nor SBW25, alone or in combination, had a significant effect on aspen seedling shoot or root biomass. The observed increase in mycorrhization cannot, then, be correlated with higher production of photosynthetic compounds from increased leaf biomass or with varying amounts of plant roots exposed to a constant fungal biomass. Therefore, the increase in mycorrhization is due solely to the presence *P. fluorescens* SBW25 and its impacts on aspen metabolism that enhance symbiosis between aspen and *Laccaria*.

Such an increase in mycorrhization may result from bacteria-induced changes in either host or fungal metabolism and their subsequent interactions (e.g., [20]) or through more complex interactions that have yet to be elucidated. The formation of ectomycorrhiza requires intricate communication and physical/biochemical interactions that bypass host immune systems designed to protect the root from pathogenic invaders and simultaneously prepare the root for colonization. Hormonal and other effectors produced by fungi stimulate the production of root primordia and alter cell wall biochemistry. The small secreted protein MiSSP7 produced by *L. bicolor*, for example, has been shown to localize in host root cell nuclei, where host jasmonic acid-responsive gene transcription is suppressed to favor ectomycorrhization [26]. In addition, the colonizing fungus may regulate the presence/reactivity of its antigenic components, such as chitin, which would reduce host immune response as well [20].

MHB may intercede in these interactions by predisposing the root for colonization. Kurth et al. (2015) [18] noted a suite of up-regulated contigs related to perception and signaling in roots of *Quercus robur*, including members of the salicylic acid and jasmonic acid signaling pathways and significant numbers of Leucine Rich Repeats (LRRs) with homology to LRR-receptor like kinases involved in recognition and signaling. Such changes would be expected to have substantial impact on the receptivity of the root to fungal colonization.

MHB may also stimulate mycorrhization by influencing fungal responses fostering colonization. For example, Labbé et al. (2014) [11] noted Pseudomonas strain-specific gene regulation in *L. bicolor* related to transcriptional regulatory complexes and biofilm formation, which may play roles in broad fungal metabolic restructuring and fungal-bacterial recognition [10]. Changes in gene expression associated with signaling pathways and fungal metabolism were also induced by *Streptomyces* in *Amanita muscaria* [21].

In the current study, we evaluated patterns of gene expression in aspen roots in dual and tripartite symbiosis to search for clues underlying the MHB relationship.

### MHB activity in SBW25 is a function of inhibition of the antifungal defense response in aspen roots

The innate immune system is constitutively expressed and is the first line of defense in plants, detecting pathogens as well as other microbes [27]. When a specific threat is detected by the innate immune system, then expression of genes specific to individual threats are activated and the innate immune system is down-regulated [28]. The proposed relationship between expression patterns is that the constitutive expression of innate immune response (Cluster 1) when aspen seedlings are cultivated alone is downregulated and exchanged for a more specific antifungal stress response (Cluster 4) when *Laccaria* is present. In order to confer MHB benefits, the antifungal systems in this cluster would be attenuated when the SBW25 is present. Among the defense responses stimulated by *Laccaria* are those involved in chitin catabolism as well as numerous ‘response to biotic stress’ genes, which reflect major potential defenses to invading fungal pathogens. When co-inoculated with SBW25, however, these responses are, by-and-large, attenuated and may reflect one of the pathways of MHB action.

Among the gene clusters identified, the ‘Global Gene Regulation’ Cluster 2 has the strongest correlation (PCC = 0.99) with observed aspen root mycorrhization and with percent RNAseq reads aligning to SBW25 (PCC = 0.77). Genes in this cluster are up-regulated when *Laccaria* is present and further up-regulated when *Laccaria* is co-cultured with SBW25. This cluster is dominated by global transcriptome reprogramming activities as well as mitochondrial function and ATP synthesis genes, which may reflect the outcome of MHB activities: increased colonization increases the demand for energy to support enhanced metabolic demand associated with supporting the mycorrhizal association.

### Analysis of gene regulatory network as Boolean circuit

The evaluation of transcriptomic data above may reflect the outcomes of interactions in the tripartite association leading to increased mycorrhization by MHB, but not necessarily the signals stimulating metabolic changes leading to enhanced colonization. While putative biological functions and the experimental conditions under which they are differentially regulated can be ascribed to co-regulated gene clusters through interpretation of expression patterns (Fig. 3) and enriched annotations (Fig. 4), we used a Boolean circuit diagram to develop a gene regulatory network that would account for the differentially regulated aspen gene clusters in the tripartite association (Fig. 5). Using this approach, Cluster 5 was identified as being the key to this regulatory network. Cluster 5 is controlled by an Exclusive Or (XOR) gate that uses the presence of SBW25 and *Laccaria* as inputs. An XOR gate is a logical operation that outputs true only when inputs differ, i.e. is activated in this model when *either* SBW25 *or Laccaria* is present, but *not* when both or neither are present. The position of Cluster 5 in the circuit and the preponderance of regulatory-related function annotations noted above (Fig. 4) makes this cluster a likely target for aspen root regulatory elements most closely associated with MHB detection and subsequent gene regulation.

### Aspen membrane-bound sensors with homology to ‘Recognition of Pollen’ proteins are candidate regulators of defense response

While the analysis of Boolean logic circuit diagram indicates that the regulation of defense response by Cluster 5 is a key component of MHB activity in this system, the specific molecular mechanisms by which detection of SBW25 by aspen roots regulates aspen root defense response gene expression are not immediately apparent. Assuming that some of the genes responsible for detecting the microbial community are differentially regulated in this experimental system, a set of specific criteria for this sensory mechanism can be hypothesized: (i) present in a cluster regulated by the presence of both *Laccaria* and SBW25; (ii) enriched in regulatory function annotated as sensors capable of integrating multiple inputs; and (iii) the regulatory gene annotations from (ii) should be uniquely enriched in the cluster identified by criteria (i).

For the first criterion, the XOR gate for the presence of SBW25 and *Laccaria* for activation of Cluster 5 is the best cluster for integrating information about the presence of community members (Fig. 5). For the second criterion, considering the sets of enriched annotations (Fig. 4), a relevant enriched annotation is ‘Recognition of pollen’ (GO:0048544). This annotation is shared by eight genes in Cluster 5 (Potri.004G028000, Potri.005G014900, Potri.010G103300, Potri.013G121000, Potri.014G086900, Potri.019G119600, Potri.019G119700, and Potri.T021600). For the third criterion, the ‘Recognition of Pollen’ annotation is indeed uniquely enriched in Cluster 5 (Additional file 4).

Recognition of pollen plays critical roles in controlling plant fertility and involves diverse molecular signatures across species. Recognition is based on small ligands (proteins, glycoproteins, lipids) from pollen that must be recognized by stigmas, with appropriate downstream metabolic responses [29, 30]. Receptors also vary extensively, including kinases, RNases, and Ca^2+^-signaling systems [30]. Given this diversity, and the potential that annotated genes in poplar may be involved in numerous aspects of recognition, the role of proteins with homology to pollen recognition in MHB interactions can be hypothesized. All eight ‘Recognition of pollen’ genes in Cluster 5 are identified as being expressed in root tissue in the DOE Joint Genome Institute database of plant gene data, Phytozome [31], which suggests both that the detection of expression in these genes in roots in this experiment is not in error and that the actual biological function of these genes in roots is not literally the ‘detection of pollen’. In the stigma, ‘Recognition of pollen’ proteins prevent accidental fertilization by incompatible pollen by integrating two separate signaling molecules present on the pollen grain to down-regulate the genes that initiate pollination [32–34]. This mechanism, i.e. integrating the information from two extracellular signals to down-regulate a suite of related genes, is precisely a match for the biological mechanism of the XOR-gated gene expression patterns in the predicted regulatory network.

## Conclusions

A model tripartite association, comprised of aspen seedlings, the ectomycorrhizal fungus *L. bicolor*, and the PGP *P. fluorescens* bacterium SBW25, was used to investigate mechanisms of MHB activity through analysis of aspen root transcriptomic data. We demonstrated that SBW25 is a MHB, promoting mycorrhization of aspen seedling roots by *Laccaria* and that SBW25 persists in the rhizosphere of sand-pot cultures after more than 60 days. We believe this to be the first report of SBW25 possessing MHB activity. A cluster of co-regulated genes in aspen roots was found to strongly correlate with the level of mycorrhization by *Laccaria*. We propose that global transcription regulation activities in this cluster, including genes for chromatin remodeling and translation activities, indicate a molecular mechanism by which aspen roots change developmental stage from free-living root to mycorrhizal symbiosis. When both *Laccaria* and SBW25 are present in the rhizosphere community, a co-regulated gene cluster annotated with plant antifungal responses is significantly down-regulated, leading to the hypothesis that MHB-mediated increase in mycorrhization in this system is facilitated through suppression of antifungal defense responses in aspen roots. A putative molecular sensor mechanism for MHB activity in roots was further identified from aspen seedlings. Eight genes with homology to proteins annotated as ‘Recognition of pollen’ are proposed as components of the root’s MHB interactions due to their patterns of expression and their annotation. These proteins can hypothetically act as a biological XOR logic gate that integrates multiple environmental/biotic signals, specifically the simultaneous presence of both *Laccaria* and SBW25 in the rhizosphere, to down-regulate the aspen root’s fungal-defense response. These model-predicted mechanisms of MHB interactions will inform the design of future biological experiments to validate our proposed molecular mechanisms of MHB activity.

## Methods

### Mycorrhizal community members

#### Aspen

Aspen and related species are some of the most widely distributed and some of the most genetically variable trees in North America [35–37]. *Populus tremuloides* (Michx.) seeds were obtained from National Tree Seed Center, Natural Resources, Canada (Seed lot # 20001017.0). The annotated aspen genome was collected from Phytozome (Ptrichocarpa210 v3.0) [31, 38].

#### Laccaria

Ectomycorrhizal fungi associate with many forest trees, such as aspen, in temperate and boreal ecosystems [35–37]. *Laccaria bicolor* (Marie) S238 N (Institut National de la Recherche Agronomique, Nancy, France) was obtained from ATCC (ATCC® MYA-4686™). The *Laccaria* annotated genome was collected from Joint Genome Institute (Lacbi2) [39, 40].

#### SBW25

SBW25 is a PGP bacterium for aspen seedlings under conditions of low nitrogen and phosphorus availability [23] and was originally isolated from the leaf surface of a sugar beet plant [9, 14, 41]. *P. fluorescens* SBW25 was a gift from Dr. Gail Preston, Department of Plant Sciences, Oxford University, United Kingdom [42]. The annotated genome was collected from NCBI (NC012660) [41, 43].

### Laboratory conditions for tripartite mycorrhizal community interactions

Four community conditions were considered in this experiment: aspen alone; aspen with SBW25; aspen and *Laccaria*; and aspen with both SBW25 and *Laccaria*. Cultures were grown in 6-cm diameter × 25-cm deep pots (Cone-tainers™, Stuewe and Sons, Corvallis, OR, USA) containing acid-washed coarse and fine sand mixture (ratio 2:1) with nylon mesh at the bottom. The experiment was performed with six biological replicates per experimental condition for a total of 24 pots.

*Laccaria* was the first community member introduced into the sand pot communities. *Laccaria* cultures were maintained on a Modified Melin Norkrans (MMN) agar medium as previously described [44]. To produce inoculum for sand pots, cultures were grown aseptically in liquid MMN medium for 3 weeks at 25 °C in the dark in static culture as described by Molina and Palmer [45]. Cultures were blended (three pulses for 3 s each) to produce a fungal slurry that was used to inoculate sand pots. To establish mycorrhizal aspen seedlings, a band of fungal slurry was added to pots and covered by ~ 2 cm of additional sand [46]. For non-mycorrhizal conditions, an equivalent volume of MMN liquid medium was added instead of fungal slurry.

Sand pots were initially planted with several sterilized aspen seeds each. Sand pots were maintained in a climate-controlled growth chamber (Caron 7305–22) with 14-h photoperiod (272 ± 31 μmol m^− 2^ s^− 1^) and day/night temperatures of 24/19 ± 3 °C; relative humidity fluctuated between 60 and 70% with temperature and time of day. Aspen seeds were kept moist by watering 3-times daily with distilled water. After 5 days, germinated seedlings were thinned to leave a single plant per pot. Following this selection, seedlings were watered three-times daily to field capacity (60 ml pot^− 1^ d^− 1^) with a nutrient solution containing 1.2 mM NO_3_, 0.4 mM NH_4_, 0.5 mM K, 0.1 mM H_2_PO_4_, 0.2 mM Ca, 0.1 mM Mg, 0.1 mM SO_4_, 50.5 μM Cl, 20 μM Fe, 20 μM B, 2 μM Mn and Zn, and 0.5 μM Cu, Na, Co, and Mo. Solution pH was adjusted to 5.6 with 0.1 N NaOH.

Five days after aspen seedlings were thinned to a single plant per pot, sand pot communities were inoculated with SBW25. SBW25 bacterial culture inoculum was grown in 20 mL of Luria Broth (LB; 10 g L^− 1^ tryptone, 5 g L^− 1^ yeast extract, 5 g L^− 1^ NaCl) medium overnight at 28 °C with shaking at 225 rpm, harvested by centrifugation at 2400 g for 20 min, and washed in the same volume of sterile 0.1 M MgSO_4_. The cell suspension was adjusted to an OD_600_ 2.0 and 150 μL were added at the base of each aspen seedling. For non-bacterial treatments, an identical volume of sterile 0.1 M MgSO_4_ was added at the base of aspen seedlings.

Tripartite communities were grown for an additional 63 days after addition of bacteria, then harvested for collection of seedling phenotype data and for metatranscriptome sequencing. Harvesting was done by gently removing aspen seedlings from pots, shaking them to remove most of the sand, and by separating roots from shoot and leaf tissues. Roots from three biological replicates per experimental condition were immediately frozen in liquid nitrogen and used for RNA extraction and transcriptome analysis. The remaining three replicates were used for measuring seedling biomass and percent root mycorrhization.

For quantitative measurement of fungal colonization, root systems were washed with sterile water and stored in sterile deionized water at 4 °C until staining. Ectomycorrhizal colonization was quantified using the gridline intersect method [47] as modified for ECM roots by Brundrett et al. [48]. Root samples from each treatment were processed by staining with 0.01% (*v*/v) acid fuchsin (Sigma-Aldrich, St. Louis, MO, USA) overnight [45] after which roots were evenly distributed in a petri dish and tips from each sample were inspected and enumerated for colonization under a dissecting microscope. Biomass measurements were done using the remaining root and shoot tissues, which were dried at 65 °C for 72 h before weighing.

### RNA extraction and transcriptome sequencing

For transcriptomic analyses, entire root systems were collected and immediately frozen in liquid nitrogen. All reagents used for RNA extraction, rRNA removal, and NGS library preparation were RNase-free molecular biology grade. Frozen plant roots were ground into a fine and uniform powder while maintained in liquid nitrogen. Approximately 100 mg of frozen root tissue was used for RNA extraction. Frozen ground root tissue was treated with 1 mL of 3x Qiagen Bacterial RNAprotect reagent for five minutes at room temperature. Treated slurry was pelleted by centrifugation at 13000 rpm for 3 min, after which the supernatant was removed from the tissue pellet. Sample was re-suspended in 450 μL of Qiagen Plant RNeasy RLT lysis buffer supplemented with fresh β-mercaptoethanol to 140 mM. The remainder of the procedure was performed according to Qiagen RNeasy kit instructions for plants and filamentous fungi, with the inclusion of an on-column DNase digest as described in Appendix D [49]. Purified total RNA was eluted in 80 μL water. Samples were purified with a Zymo Research OneStep PCR inhibitor removal desalting column with elution into water. Yield and quality of desalted sample was assessed using the Nanodrop and an Agilent Bioanalyzer chip run on the plant RNA protocol. A second DNase treatment was performed on one to three micrograms of total RNA sample using the Epicentre Baseline-Zero DNase enzyme at 0.3 to 1.0 units of enzyme activity per μg of total RNA for 15 min at 37 °C. DNase activity was stopped by immediate purification using a Zymo Research RNA Clean & Concentrator-5 column with elution into 20 μL of water. Concentrated total RNA was depleted of rRNA subunits using a 50–50% mix of Plant Seed/Root and Bacteria rRNA probes Epicentre/Illumina Ribo-zero probes. The resulting mRNA sample was purified using Agencourt RNAClean XP magnetic beads according to kit instructions and eluted in 12 μL water. Successful removal of the majority of rRNA subunits was confirmed and sample quantified using an Agilent Bioanalyzer Pico RNA chip run on the mRNA protocol.

Strand-specific NGS transcriptomic libraries were produced from sample mRNA using the Epicentre Script-Seq kit according to kit instructions. Each library was appended with a unique barcode to enable sample multiplexing and amplified for 18 cycles using the recommended Fail-safe polymerase and buffer (Epicentre). Libraries were purified using Agencourt AMPure XP magnetic beads according to kit instructions and eluted into 16 μL of water. Completed libraries were characterized and quantified using an Agilent HS DNA chip and Qiagen HS DNA Quant-it reagent. NGS library sequencing services were provided by the Institute for Genomics & Systems Biology HGAC sequencing core at the University of Chicago. Up to six libraries were multiplexed per Illumina HiSeq200 flow cell and sequenced on a 50 bp cycle sequencing protocol.

### Transcriptomic data analysis

#### Generate metatranscriptomic data from sequencing reads

Gene expression data for the plant-symbiont communities was determined from RNAseq reads. Bowtie [50] was used to align short read sequences to community (aspen, *Laccaria*, and SBW25) gene models (i.e. predicted coding sequences). Gene models for all community members were collected from the public databases listed above. The default Bowtie conditions were used to generate alignments for all sets of sequence reads to gene models, except for setting Bowtie to return all possible sequence alignments. Gene model expression was detected in the collected transcriptomics data using the application BowStrap [24]. 10,000 BowStrap iterations were used for the calculation of average and standard deviations of Reads Per KBase gene per Million aligned reads (RPKM) values.

Significance of gene expression was determined using a Cumulative Normal Distribution (CND) based *p*-value, adjusted by Benjamini-Hochberg (BH) false discovery rate (FDR) correction [51]. A gene was considered significantly expressed with a BH-corrected p-value less than 0.05 and bootstrapped RPKM expression level greater than 2. All gene expression values were normalized by Quantile Normalization [52].

#### Cluster significantly differentially expressed genes by patterns of co-regulation

Significantly differentially expressed (SDE) genes were identified by 2-factor analysis of variance (ANOVA) using MeV 4.5.1 (https://sourceforge.net/projects/mev-tm4/). The factors considered for ANOVA were presence or absence of *Laccaria* and presence or absence of SBW25. *P*-values were calculated based on 10,000 permutations with a significance threshold of less than 0.05.

All SDE genes were grouped into clusters of co-regulated genes using K-means clustering. Clustering was performed considering Euclidian distances and calculated using ‘R-project’ (v 3.0.3) (https://www.r-project.org/). The optimal number of clusters considered was determined using Silhouette coefficients, evaluating all cluster sizes between 2 and 12 and selecting the cluster size that provided the largest Silhouette coefficient [53].

#### Identify enriched gene annotations in gene subsets

To place the clusters of genes into a broader biological context, the specific Gene Ontology Biological Process (GO-BP) [54] annotations that are enriched in gene clusters, relative to the distribution of annotations in the total annotated aspen genome, were identified. Enrichment for a GO-BP annotation for a given datatype was calculated as a Cumulative Hypergeometric Distribution [55]. A threshold of *p*-value less than 0.05 was used to determine significance of GO-BP annotation enrichment.

#### Generate aspen root MHB regulatory network

To generate a network of causal interactions between clusters of co-regulated genes, Bayesian Network (BN) inference was used. For each replicate community transcriptome, the average gene expression for genes within a K-means cluster was calculated. In addition to average gene cluster expression, nodes for the presence/absence of *Laccaria* and SBW25 were considered. BN was generated using BANJO [56, 57] with the following parameters: greedy search algorithm, all local moves, and a maximum of 5 parents per node. In addition, nodes for ‘*Laccaria*’ and ‘SBW25’ were not permitted to be the child of any other node. The resulting highest-scoring network was then pruned of redundant interactions (e.g., if the BN identified the causal interactions A → B, B → C, and A → C, then the interaction A → C was removed from the network).

The pruned BN was then used as the scaffold of a logic diagram, implemented by Boolean circuits, where the output of MHB activity is a function of the presence or absence of *Laccaria* and SBW25 only. The selection of logic gates is the result of a careful interpretation of the data. While Boolean relationships cannot capture the entire possible range of differential gene regulation, this network effectively summarizes a regulatory interaction network that condenses the relationships between gene clusters in the data into a single graphical representation.

## Additional files

Additional file 1: Table of RNAseq alignments. Table presents total number of reads per sequencing reaction, number of reads that align to each community member, and the percent of total aligned reads that align to each community member. (XLSX 9 kb)

Additional file 2: RPKM scores for all samples. This file includes results from Bowstap analysis of transcriptomic data for aspen gene expression and ANOVA results. Data types are ‘Unique RPKM’ which disregards sequence fragments that align to more than one gene, ‘Ave RPKM’ which is the results including bootstrapped analysis including multiply-aligning sequences, ‘SD RPKM’ which is the standard deviation of bootstrapped alignment RPKM values, ‘Log2Norm RPKM’ which is the normalized gene expression values, ‘pVal’ which is the significance of gene expression calculated from Bowstrap analysis, and ANOVA-pVal which is the significance of analysis of variance by presence of *Laccaria* (LBI), presence of SBW25, and the interaction condition. (CSV 22927 kb)

Additional file 3: Annotated transcriptome, grouped by expression cluster. Data is presented as an Excel file with a worksheet for each cluster of co-regulated genes. In each worksheet, gene ID, gene expression (normalized RPKM), significance by ANOVA, Log2 fold change relative to aspen root only condition, and gene annotations are given. Annotation data includes links to additional gene information in ‘Phytozome’ and ‘The Arabidopsis Information Resource’. (XLSX 2256 kb)

Additional file 4: Table of enriched GO-BP annotations by cluster. Enriched BP-GO annotations are grouped by co-expressed gene cluster. Numbers in parenthesis after each annotation term in the number of genes with that annotation in the cluster. (XLSX 9 kb)

Additional file 5: Bayesian Network for Gene Cluster interactions. The initial Bayesian Belief Network used to construct the Boolean circuit for gene regulation interaction in Fig. 5 is presented as a list of target-parents. Note that the Bayesian network was trimmed as described in Methods prior to development of Boolean circuit. (TXT 367 bytes)

## Abbreviations

BN: Bayesian Network
CND: Cumulative Normal Distribution
FDR: False Discovery Rate
GO-BP: Gene Ontology Biological Process
MHB: Mycorrhizal Helper Bacteria
MMN: Modified Melin Norkrans
PGP: Plant Growth Promotion
RPKM: Reads Per Kilobase of transcript, per Million mapped reads
